# Understanding Disease Control: Influence of Epidemiological and Economic Factors

**DOI:** 10.1371/journal.pone.0036026

**Published:** 2012-05-09

**Authors:** Katarzyna Oleś, Ewa Gudowska-Nowak, Adam Kleczkowski

**Affiliations:** 1 M. Kac Complex Systems Research Center and M. Smoluchowski Institute of Physics, Jagiellonian University, Kraków, Poland; 2 Department of Computing Science and Mathematics, University of Stirling, Stirling, United Kingdom; University of Zaragoza, Spain

## Abstract

We present a model of disease transmission on a regular and small world network and compare different control options. Comparison is based on a total cost of epidemic, including cost of palliative treatment of ill individuals and preventive cost aimed at vaccination or culling of susceptible individuals. Disease is characterized by pre-symptomatic phase, which makes detection and control difficult. Three general strategies emerge: global preventive treatment, local treatment within a neighborhood of certain size and only palliative treatment with no prevention. While the choice between the strategies depends on a relative cost of palliative and preventive treatment, the details of the local strategy and, in particular, the size of the optimal treatment neighborhood depend on the epidemiological factors. The required extent of prevention is proportional to the size of the infection neighborhood, but depends on time till detection and time till treatment in a non-nonlinear (power) law. The optimal size of control neighborhood is also highly sensitive to the relative cost, particularly for inefficient detection and control application. These results have important consequences for design of prevention strategies aiming at emerging diseases for which parameters are not nessecerly known in advance.

## Introduction

The network-based approaches are a common tool in epidemiological studies [1]. These individual-based methodologies allow incorporating the diverse patterns of interaction that underlie disease transmission and have been proved to capture topology of populations [2], [3]. An interesting aspect of such studies, with an obvious goal to target spread of the disease, is identification of optimal strategies for the control of a disease under additional constraints [4]–[6]. Network modelling has been successfully used for many systems in order to design such control strategies [7]. However, there are only very few attempts to incorporate economic factors in such realistic models. Conversely, bioeconomic models usually ignore the spatial components of the disease spread [8]–[10].

In this paper we present a combined epidemiological and economic model to address the problem of optimization of disease control on networks with incomplete knowledge. Two main sources of costs can be associated with a disease outbreak and its control: the palliative cost associated with disease case and costs of measures aimed at preventing further cases [11], [12]. The objective of preventive actions is to lower the total cost by investing e.g. in vaccination at the initial stages of the epidemic or culling of infected/susceptible individuals.

In our approach, we define a measure of the total cost of the epidemic (the severity index, *X*) and analyze the influence of the parameters on its minimum. Work so far has shown that it is possible in such models to find an optimal control strategy [12]. Three optimal control scenarios (Global Strategy (GS), Local Strategy (LS), Null Strategy (NS)) emerge from the cost-effectiveness analysis. However, the relationship between the details of the Local Strategy and the model parameters is still elusive [7], [12]. Establishing such a relationship is an essential step in designing control strategies for emerging diseases and hence we have concentrated on this task in the paper. We investigate propagation of the disease in a small-world network. The basic topology represents a regular lattice, with additional long-range bonds between randomly chosen pairs of sites. Inclusion of shortcuts into a regular lattice enhances communication of the disease and causes proliferation of epidemics at locations far apart from the original infected source.

Our principal objective is to identify optimal strategies for eradication of the disease by determining the threshold size of the control neighborhood. In the proposed model, the neighborhood order *z* is introduced as a measure of either the distance that the disease can spread (epidemic neighborhood), or the spatial extension of the control measures in a single “event” (control neighborhood). To investigate how limited resources should be balanced between disease detection and eradication, we analyze combined effects of the average time until detection and the treatment rate on optimal control size of the neighborhood.

We have found that the scale of control matches the scale of dispersal of a disease and so the larger the infection neighborhood, the further the control has to be extended. This relationship can be approximated by a linear function which coefficients depend algebraically on the detection and treatment rates following a power law. Small change in the relative cost of preventive to palliative treatment may result in big changes in this relationship. Addition of small world links narrows the range where the scaling (power) law is valid but the scaling persists for small values of detection and treatment times.

## Methods

### Model

We assume that individuals are located at nodes of a regular (square) lattice that represents geographical distribution of hosts. On this lattice, we define a local neighborhood of order *z* as a von Neumann neighborhood in which we include *z* shells and 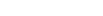 individuals, excluding the central one. Accordingly, 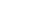 corresponds to a single individual, which means that this individual is not in contact with anyone, 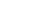 corresponds to 4 nearest neighbors while 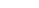 corresponds to the whole population in the limit of infinite size of the system. For the small world model a fixed number of long range links has been added to the regular network described above. Those links span the whole population, but otherwise behave like local links.

**Figure 1:**
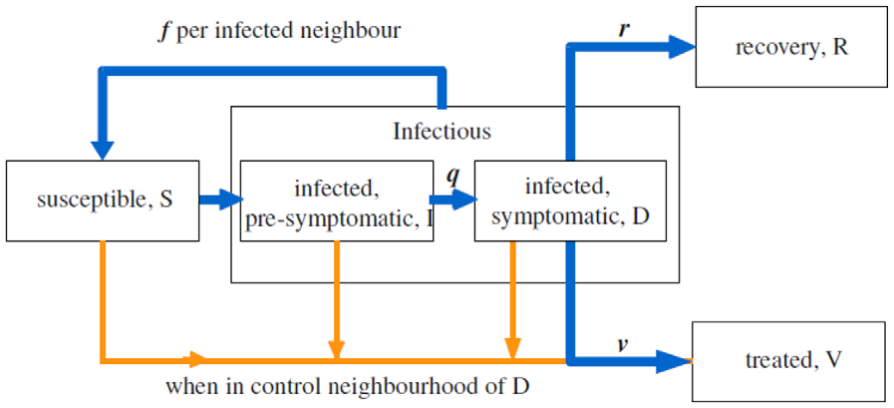
Block diagram illustrating transitions in the model: transitions performed at each time step (blue solid lines) and transitions triggered by treatment (orange thin lines).

The epidemiological model is a standard SIR (Susceptible-Infected-Removed) model [13], modified to include pre-symptomatic and symptomatic stages of the disease and to account for detection and treatment (cf. fig. 1). All individuals are initially susceptible (**S**) and the epidemic is initiated by introduction of several infected (**I**), pre-symptomatic individuals. Each of infected individuals (symptomatic and pre-symptomatic) stays in contact with a given (fixed) number of other individuals in its infection neighborhood of order 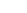 After infection, the susceptible individual moves first to infected, pre-symptomatic class, (**I**) compartments. It can further infect its neighbors with probability *f* per a contact but cannot be treated yet. As symptoms develop with probability *q*, individual moves to **D** class and can be detected. It is still infectious but can spontaneously recover with probability *r* and accordingly, move to a recovery class, (**R**) and cannot be further infected or treated.

Detection triggers the control process which becomes activated with probability *v*. In consequence, all individuals (except **R**) within control neighborhood of size *z* centered at the detected host, transfer to the treated class **V**. The order of control neighborhood *z* may be different from the order of infectious neighborhood 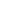 and is typically larger. Accordingly, the group of individuals subject to the treatment is composed of at least one symptomatic and a mixture of susceptible and infected pre-symptomatic individuals. For convenience, we extend the definition of the neighborhood *z* to capture situations when no spatial control is applied (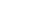), or when the treatment is applied solely to the detected individual (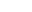).

Numbers of individuals in each class are denoted by *S*, *I*, *D*, *R* and *V*, respectively with *N* = *S+I+D+R+V* being the total constant number of individuals in the population.

In order to investigate the optimal control strategy, we need to compare value of future benefits (reduction of infection cases) with the value of future and current costs associated with a particular choice of measures in disease control and treatment. In this paper we allocate the costs to two groups:
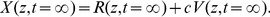
(1)


The first term represents the palliative cost and is associated with individuals who are not treated and therefore spontaneously move into the **R** class. The second term describes costs associated with treatment of detected individuals and their neighbors and is assumed to be proportional to the number of treated individuals *V*. In the above formula *c* represents a cost of treatment relative to the cost of infection and *z* stands for the control neighborhood size. Both estimates of *R* and *V* are evaluated at the end of each simulation run (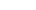).

### Simulations

Monte Carlo simulations have been performed on a regular grid of 200 by 200 cells with periodic boundary conditions with and without long-range links. This choice of size has been dictated by a trade off between numerical efficiency and avoidance of small-size effects which could influence results. Additional numerical tests proved the consistency of results for different system sizes [12].

**Figure 2:**
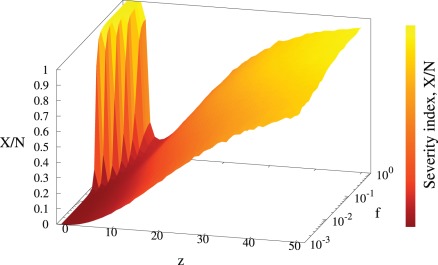
Severity index, X, as a function of the infection rate per contact *f* and the control neighborhood size *z*. Simulation parameters: 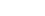, 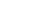 with 40 initial foci and infected neighborhood size set to 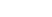 cost 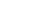

Epidemics have been initiated by addition of 40 infected individuals to an otherwise susceptible population. Each simulation run has been continued until 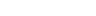 (i.e. up to the time when no further infection can occur). Subsequently the severity index *X* has been evaluated from the formula eq.(1). The optimal strategy is then determined by the minimal value of the severity index 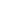 The corresponding value of *z* gives the optimal size of the control neighborhood, 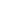 (see fig. 2 for illustration). In the simulations, the minimization of the severity index is achieved by sweeping through different values of the control neighborhood size *z*, while keeping other parameters fixed. For each value of *z* only a single simulation has been performed. Collections of this results yield a dependence of *X* on *z*. A minimum value of *X* in this collection gives an estimate of 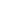 and the corresponding *z* gives an estimate of 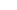 This procedure has been repeated 100 times to yield representative average values of 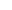 and 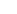 and their corresponding standard deviations.

## Results

The long time (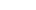) behavior of the model in the absence of control (Null Strategy, NS, i.e. 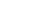) is determined by the probability *f* of passing the infection to a susceptible node from any of its neighbors within the neighborhood size ranging from 4 (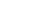) to 144 (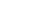). For small *f*, the infection quickly dies out. Disease spreads invasively over the population for large *f*, when no control is applied, 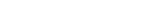 When 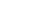 the ratio 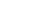 declines with the order of the control neighborhood. However, at the same time the number of treated individuals *V* increases, contributing to the total cost *X*, cf. eq.(1). For 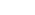 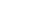 is either a monotonic function of *z* for small values of *f* or a non-monotonic function for highly contagious disease (large *f*), see fig. 2.

**Figure 3:**
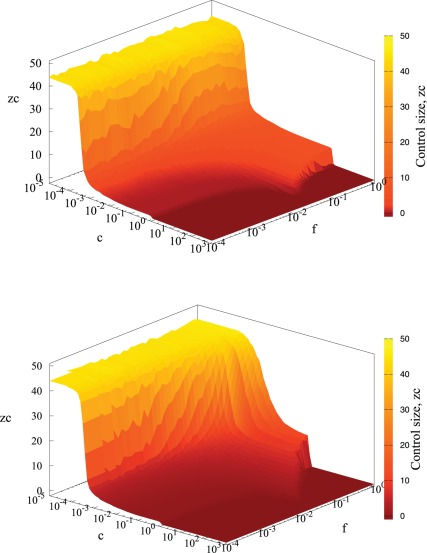
Control neighborhood size as a function of treatment cost *c* and infectiousness of the disease *f* for regular network and small world model. Simulation parameters: 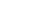 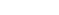 with 40 initial foci and 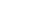 Control size 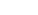 represents local strategy (LS), 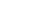 corresponds to the strategy when only the detected individual is treated and 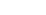 denotes GS (more than 99% of individuals are treated). Null strategy corresponds to 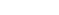 Top figure denotes results for disease spreading on regular networks, whereas bottom to small world model with inclusion of additional 2000 number of long range links (5%).

Three regions can be identified in the dependence of 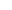 on *c* and *f*, see fig. 3. For small values of *c*, Global Strategy (GS) is dominating, whereas for large *c*, it is best to refrain from treatment, Null Strategy (NS), fig. 3.

Although the location of the minimum of 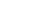 varies with increasing *f* and *c* values (see figs. 2, 3), a relatively wide plateau region with an almost constant 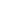 develops for intermediate values of *c* and *f* and corresponds to the local strategy (LS), fig.3. The structure in fig. 3 is partially deformed by addition of long-range links, however, the plateaux persists for small values of *f*.

**Figure 4:**
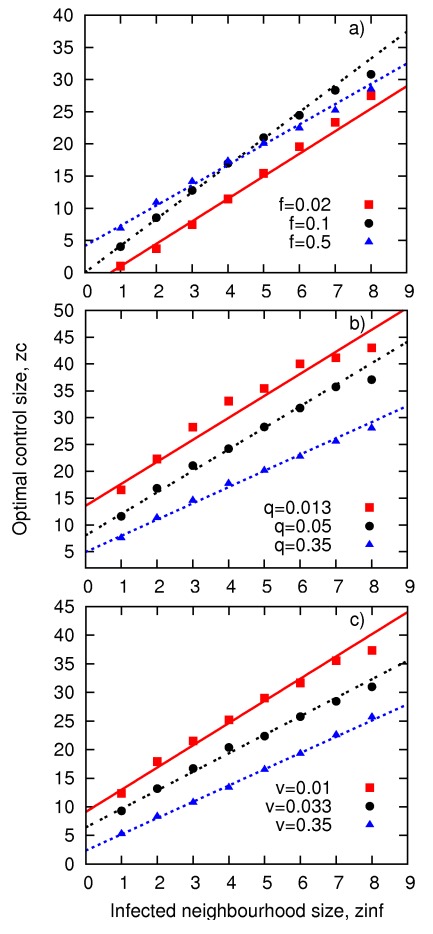
Relationship between 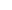 and 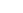 **for treatment cost** 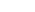. Points mark the simulation results whereas lines correspond to fitted linear function 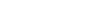 From top to bottom, the following sets of constant kinetic parameters have been assumed: (a) 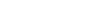 (b) 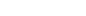 (c) 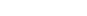 Errors (standard deviation from the mean) are too small to be visible.

We have therefore focused on the plateaux region (LS) of 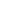 and have explored its dependence on epidemiological parameters: 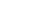 with constant *f* and *c*. We have first explored dependence of 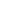 on the size of infection neighborhood for 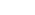 see fig. 4. The relationship can be accurately approximated by a linear function for a wide range of parameters, infectiousness *f* (fig.4a), the rate at which symptoms appear, *q* (fig.4b) and the treatment rate, *v* (fig.4c) for 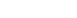


As already seen in fig. 3, infectiousness *f* hardly affects the slope and intercept of the linear relationship, fig.4a. Increasing *q* and *v* causes the lines to shift towards lower values of 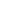 with major changes in the intercept but slope only slightly affected (cf. fig.4b,c). In contrast, the relationship between 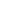 and *q* (or *v*) for fixed 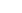 is non-linear. It is more convenient to consider 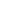 instead of *q* as 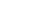 has an interpretation of average time till detection of symptoms. Similarly, 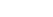 can be interpreted as an average time till treatment.

**Figure 5:**
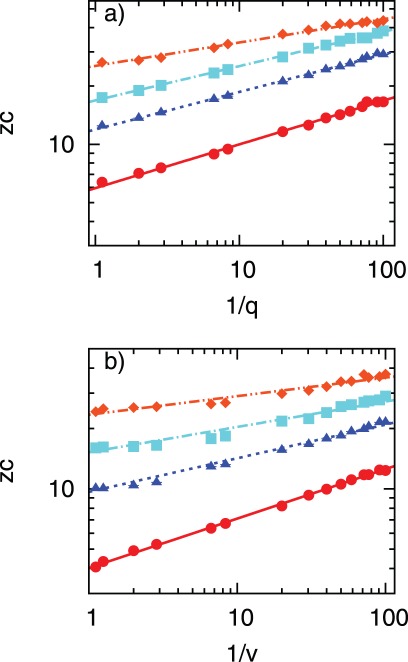
Relationship between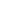 and 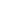 in a) and 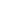 in b). Points mark the simulation results and lines correspond to fitted functions: a): 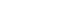 and b): 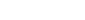 for red: 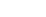, navy blue: 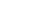 blue: 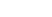 orange: 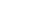

Broadly speaking, 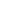 increases with 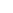 and 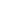 fig. 5. This is consistent with the following mechanism. Consider a single infected but pre-symptomatic individual. The disease focus centered on it will spread until appearance of symptoms after time 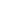 Thus, the longer it takes to discover symptoms of the disease, the farther the disease would spread from its original focus. As a consequence, the infected area becomes larger and so does 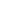 Similarly, the longer time from detection until treatment, the further the disease moves away from original focus. As a result, the control size grows with increasing treatment time.

Intriguingly, it appears that 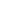 scales algebraically with 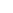 (and with 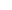) following a power law: 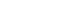 and 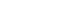 eq.(3) (see fig. 5) with exponents well below 1.

The exponents 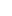 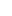 are similar for a range of 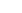 within the plateaux regime of an optimal control radius of the epidemic, (see fig.3), i.e. for 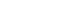 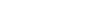 and 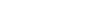.

**Figure 6:**
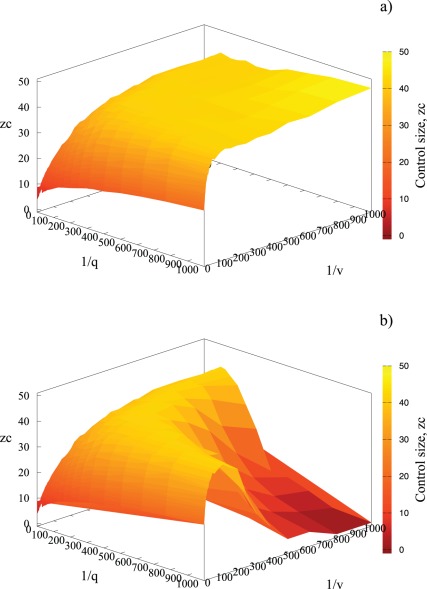
Control neighborhood size as a function of both detection time, 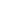 **and recovery time,** 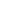 for 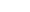 in a) and 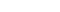 in b). Simulation parameters: 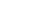 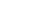 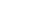 40 initial foci.

While fig. 5 is representative of results for 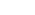 moving *c* just beyond 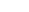 causes a dramatic change in the 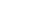 dependence for large values of 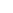 and 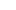 corresponding to detection and vaccination time comparable with duration of epidemics (approximately 10^4^ time steps for large values of 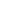 and 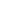). The control neighborhood 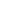 decays abruptly for increasing times 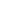 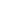 as illustrated in fig. 6. This change is associated with very inefficient control (long time till detection, 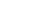 and long time from detection to treatment, 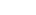). If the cost of control is lower or equal to the cost of palliative care, it is still better to treat, even though we are not very efficient with treatment and most individuals are spontaneously removed. However, if the cost of vaccination is only marginally higher than the cost of untreated case, prevention is no longer cost-effective. We also note that it is only a combination of very long values of 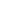 *and* 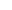 that leads to a limited range of application of the scaling formulas (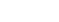 and 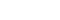).

**Figure 7:**
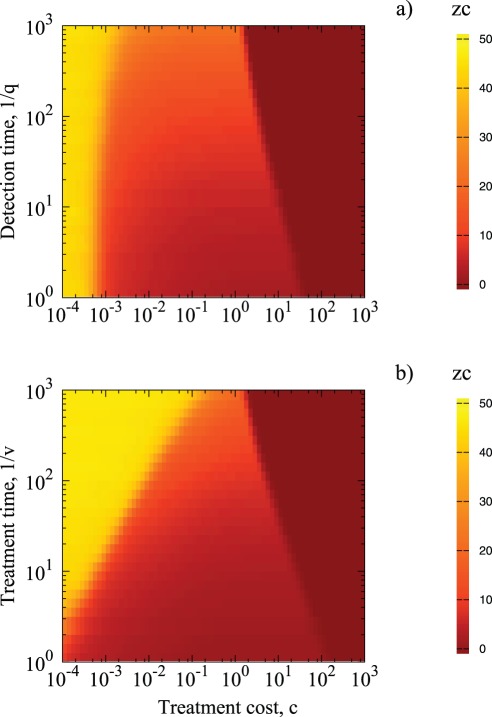
Control neighborhood size as a function of treatment cost *c* and detection time 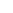 (a) and treatment time 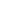 (b). Simulation parameters: 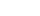 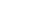 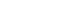 with 40 initial foci and 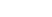 Color borderlines between different regions indicate transition regions among various optimal strategies.

The scaling region of 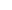 as a function of 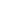 and 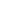 also depends on *c* in a fashion reminiscent of fig. 3. For small values of *c*, Global Strategy of treating everybody is optimal regardless of the parameters, cf. fig. 3 with fig. 7. In contrast, Null Strategy is optimal for large *c* (figs. 3 and 7). The region where Local Strategy is optimal occupies the region near 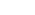 but it becomes narrower when the disease is more infectious (fig. 3) or when the control is less efficient (for increasing values of 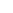 (fig. 7a) and 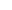 (fig. 7b). Within this region, 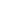 is given by scaling formulas. As seen before, 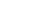 is a special case asymptotically associated with a breakdown of LS for very large or very small *f* (fig. 3) and very large values of 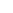 and 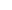 (fig. 7).

**Figure 8:**
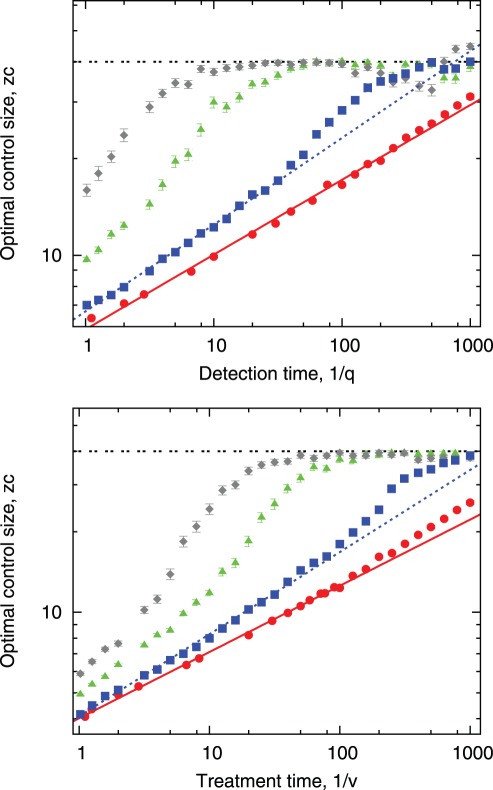
Relationship between 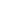 and 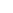 **on top and** 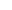 on bottom for regular and small world network with varying number of long-range links. Lines and simulation points from bottom: red: regular network, navy blue: small world with additional 1% of links, green: small world with additional 5% of links, grey: small world with additional 10% of links, dashed black: 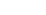 which corresponds to treating the whole population. Simulation parameters: 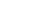 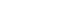 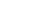 40 initial foci, 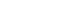 of long-range links.

The addition of long range links shitfs the optimal radius of control towards larger values, figs. 3, 8. The scaling behaviour (cf. fig.5 ) is characteristic for a regular network and changes when long-range bonds is added (see fig.8). With 400 random long-range contacts (corresponding to 1% of all links) the scaling relation between 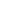 and 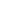 (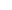) breaks down for detection (treatment) times exceeding 10. This is clearly indicated by deviation of the results from red bottom line (in fig.8) denoting simulation data for regular networks (the same as in fig. 5). Altogether, addition of small world links reduces the range of detection 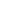 and treatment 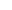 times for which the power law relationship is valid. This is caused by long range links allowing disease to escape from the local control. In contrast, if we are able to detect disease quicker, it has not much chance to escape and the disease spread is effectively short range. Consequently, the scaling can be observed for small values of detection and treatment times, 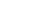 In summary, with increasing degree of randomness of networks (larger number of links) not only the control radius rises but also the scaling disappears. Note that the dashed black line, 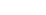 in fig. 8, represents Global Strategy.

## Discussion

In order to design a successful strategy for controlling a disease we need to take into account not only epidemiological and social factors (including the topology of the social network of contacts and in particular 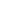), but also economic considerations. Some of these factors might be unknown or hard to estimate, particularly in real time as the epidemic unfolds. It is therefore crucial to understand the relationship between the optimal control strategy and parameters, for a wide range of possible values. It is even more important to establish those processes and parameters to which a selection of optimal strategy is not particularly sensitive, as this allows us to find strategies that can be designed in advance, even without knowing their actual values for a given emerging disease.

Regular networks have been traditionally used for modelling epidemic outbreaks of human, animal and plant diseases [14], [15] and many variants of such an approach (with e.g. constant or randomized probabilities of infection passed to neighbouring nodes on a grid) have been studied. However, an accumulated experimental evidence demonstrates that real systems rarely follow this kind of idealization being neither completely random nor located on regular lattices. Among other types of networks that have been the object of intense studies are the small-world and scale-free networks. In particular, the small world network with randomly chosen shortcuts between the nodes, is considered a model well extrapolating between extremes like regular and random network. It has been also preferentially used by modellers discribing outbreaks of disease starting simultaneously in different regions of the world (propagation of the SARS virus, [16]. Accordingly, in order to assess the occasional long distance dispersal of the disease, we have also considered small world links, representing e.g. random transport by wind or by plane.

In our previous paper we have shown that for a given set of 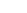 *q* and *v*, the broad choice of the strategy is determined by the relative cost of the treatment, *c*. For small values of *c*, GS is optimal, for large values of *c*, NS. Close to 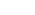 a LS dominates and the detailed value of the control neighborhood 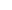 depends on the epidemiological parameters, although not on *f* in a wide range. In this paper we extend this analysis to include other epidemiological parameters. In particular we show that the broad division between GS (for 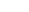), NS (for 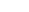) and LS (for 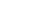) holds for a wide range of parameters *q* and *v* (inverse of time to detection and inverse of time to treatment, respectively), fig. 7.

Three other key results emerge from our analysis. Firstly, it is very important to match scale of control to the scale of infection dispersal. This has already been seen in other papers [17], but this is the first time we show it for spatial control on networks in the presence of economic evaluation. However, we also show that the size of the control neighborhood is not just simply equal to the size of the infection neighborhood (see fig. 4 and compare the scale of horizontal and vertical axes). In the presence of pre-symptomatic individuals (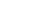) and in the face of delays associated with application of control (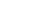) we need to extend 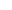 well beyond 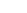 The relationship between 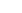 and 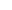 is one of the key formulas for planning response to epidemics. It enables authorities to plan actions aiming at eradication of the disease by setting a sufficiently large – but not too large – zone of eradication around each detected case. Traditionally, such recommendations are based on the dispersal patterns of the disease, although increasingly simulation models are used. This procedure has led to establishment of the 1,900ft rule for citrus canker [18] whereby all citrus trees are cut down within this radius from every affected tree and the 3 km/10 km rule for foot-and-mouth disease [19].

However, our results show that the relationship between 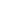 and 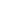 is non-trivial and in particular it involves non-linear functions of 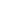 and 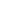 Although we are still far from being able to provide a formula relating 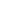 to all epidemiological parameters, our result stresses importance of using models to design control strategies [20].

We also show that 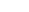 is a special case. In particular, we show high sensitivity of 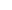 to changes in *c* for large values of 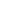 and 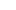 Thus, if the symptom detection time (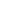) and reaction time (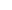) are both long, small change in *c* leads to very big changes in 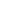 see fig. 6 and 7. Without knowing the exact value of *c* it is therefore very difficult to design the strategy in this case. Suppose we believe that 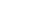 and therefore we chose a small value of 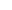 based upon fig. 6b. However, if in reality 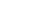 (although very close to 1), 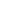 should be close to 50 (fig. 6a). This shows the importance of knowing what the actual value of *c* is [12] estimated that for vaccination 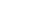
–0.85, but can be larger than 1 for culling.

In this paper we have used regular and small wold networks to describe the topology of interaction between individuals. Addition of small world links into population narrows the range where the scaling (power law) relationship of 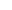 on 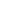 and 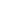 is valid but the scaling persists for small values of detection and treatment times.

Our studies can also be extended in other ways. The current work assumes relatively short overall time length of each epidemic and so no discounting is applied when the costs and benefits are estimated. We also assumed that the strategy is unchanged throughout the epidemic and that the network structure is static and relatively simple. Each of these assumptions can be relaxed. Discounting is often used in economics, but we expect for it to have a small impact on our results. Adapting the strategy to the current status of the epidemic often leads to a bang-bang solution [21], similar to our distinction between NS and GS.

Finally, a lot of attention have been recently given to non-local and random networks (small-world or scale-free networks) [12], [22], to dynamic networks [23], and networks with random parameters [24]. Further extension of this work to include static and dynamic disorder is in progress.
